# Cost-effective synthesis of 2D molybdenum disulfide (MoS_2_) nanocrystals: An exploration of the influence on cellular uptake, cytotoxicity, and bio-imaging

**DOI:** 10.1371/journal.pone.0260955

**Published:** 2022-01-18

**Authors:** Dhirendra Sahoo, Sushreesangita P. Behera, Jyoti Shakya, Bhaskar Kaviraj

**Affiliations:** 1 Department of Physics, School of Natural Sciences, Shiv Nadar University, Uttar Pradesh, Greater Noida, India; 2 Faculty of Life Sciences and Biotechnology, South Asian University, New Delhi, India; 3 Department of Physics, Indian Institute of Science, Bangalore, India; Mohanlal Sukhadia University, INDIA

## Abstract

Ultrasmall MoS_2_ nanocrystals have unique optoelectronic and catalytic properties that have acquired significant attraction in many areas. We propose here a simple and economical method for synthesizing the luminescent nanocrystals MoS_2_ using the hydrothermal technique. In addition, the synthesized MoS_2_ nanocrystals display photoluminescence that is tunable according to size. MoS_2_ nanocrystals have many advantages, such as stable dispersion, low toxicity and luminescent characteristics, offering their encouraging applicability in biomedical disciplines. In this study, human lung cancer epithelial cells (A549) are used to assess fluorescence imaging of MoS_2_ nanocrystals. MTT assay, trypan blue assay, flow cytometry and fluorescence imaging results have shown that MoS_2_ nanocrystals can selectively target and destroy lung cancer cells, especially drug-resistant cells (A549).

## Introduction

Few-layered MoS_2_ nanocrystal, one of the typical two-dimensional (2D) transition metal dichalcogenide materials, show the unique mechanical, optical, electrical, and chemical properties correlated with their ultrasound atomic layer structure and tendering them an appealing alternative to fluorescent dyes, and have attracted particular attention in the scientific uses. MoS_2_ nanocrystals can be used in many biomedical applications because of their tunable size and adequate luminescence properties. It has been widely applied in medicine, drug delivery, diagnostics, and outstanding biocompatibility in living organisms [1,2].

Lung cancer is the leading cause of cancer patient fatalities in the United States. And throughout the world. Nearly as many Americans die from lung cancer each year as prostate, breast and colon cancers combined. Chemotherapy has always been one of the most common ways to treat cancer over the last few decades. However, chemotherapy creates some remedial barriers, such as serious side effects, low solubility and a tendency to drug resistance [3–5]. However, the trade-in nanotechnology is growing rapidly, and nanoparticles apply to a variety of areas of our real-world applications [6]. For this reason, the possibility of communication of individuals with different types of nanoparticles is also underway. For this purpose, it is essential to study how nanoparticles can influence the human body because they can do so through respiration, skin contact or ingestion [7]. Despite the fact that nanocrystals have been produced and applied for several decades. Their impact on health and the environment has not been fully explored due to the complexity of how nanocrystals and their ingredients interact with cells [8].

The challenges of producing the synthesis and versatility of nanocrystal compositions and a wide spectrum of the available surface ligand still exist. A number of nanostructures, including carbon nanotubes, graphene, fullerenes and quantum dots, have been synthesized because they demonstrate the encouraging potential to overcome the shortcomings of chemotherapy drugs for cancer treatment. Corresponding to specific nanoparticles, two-dimensional (2D) nanoparticles have unique chemical, optical and electronic characteristics and are therefore provided with unique healing tools for biomedicine, particularly cancer treatment [9]. The bulk MoS_2_ contains multilayered arrangements with weak van der Waals force of attraction between layers and strong S-Mo−S interlayer covalent bonding. This allows for easy isolation of a single layer of MoS_2_ of bulk crystals. Therefore, mechanical exfoliation, electrochemical intercalation, liquid exfoliation, ultra-sonication and were investigated to produce a single- or few-layer MoS_2_. These methods also lack low productivity, complexity and time. In addition, synthetic MoS_2_ production should be monitored to understand the maximum production yield. Thus, it is necessary to produce new approaches to the production of layered MoS_2_ with the tunable size to investigate the implications of emerging applications [10,11].

Some important studies show the promising potential application of 2D nanoparticles in targeted cancer control [8,12,13]. An additional effort has been made to look for other similar 2D materials in relation to distinctive unique properties. The MoS_2_ nanoparticle, like a variety of metallic transition dichalcogenides (TMDC), has demonstrated potential applications in nanoelectronics, energy storage devices, and electrochemical storage, catalysis, biomedical science, and diagnostic applications. Despite remarkable progress in MoS_2_ nanoparticle synthesis, it is necessary to find out a facile approach to produce MoS_2_ nanocrystals with strong fluorescence. MoS_2_ is an excellent material with high dielectric, thin and highly available surface area that constantly increase the path of light propagation in the sample. It also shows the considerable formation of surface defects having Mo and S vacancies during the synthesis process and acts as dipoles under the irradiation of light to boost interface polarization and defect dipole polarization for more attenuation of light. In recent years, the synthesis and use of atomically thin MoS_2_ nanocrystals has received considerable attention in materials research [14–16].

The synthesis and treatment of MoS_2_ nanocrystals for the viability of A549 cancer cells is demonstrated here. This work aims to show an eco-friendly, facile and reproducible synthesis method based on a hydrothermal process for the scalable production of MoS_2_ nanocrystals (2–10 nm). This work explains the preparation of thin-layer MoS_2_ nanocrystals using a single-step hydrothermal method using sodium molybdate and thioacetamide as sources of molybdate and sulfur, respectively. Cost-effective and facile approaches to controllable synthesis of MoS_2_ nanocrystals are quiet in critical demand, and the potential biomedical application of these MoS_2_ nanocrystals should be developed. As the study of the TMDs, nanomaterial toxicity is still in its origin with hardly a few assessments conducted on a mono or few-layer TMDs (e.g., MoS_2_, WS_2_). It is not surprising that no consistent research has yet been conducted to detect the toxicity of TMDs. It is necessary to start studying the toxicological consequences of nanomaterials to indicate the health risks they can claim. It is fairly well researched that bulk TMDs have low toxicity. Yet their research on nanostructures is still inadequate and poorly understood. The chemically exfoliated layers of MoS_2_ nanosheet are more toxic, which is due to the increase in surface area. Low toxicity of MoS_2_ and thin WS_2_ was observed from their cellular evaluations using MTT and water-soluble tetrazolium salt (WST-8) analyses on A549 cells [13,17–23]. The toxicity of the MoS_2_ sample is also caused by the organic solvents used in chemical exfoliation. It has been an impediment to the accurate analysis of its toxicity for a duration. However, it is difficult to synthesize the thin layer MoS_2_ without chemical exfoliation. Atomically thin MoS_2_ films prepared using mechanical exfoliation and chemical vapor deposition methods yield very less amount than sufficient for biological testing [24–29]. Jun Lou et al. confirmed the low toxicity of molybdenum disulfide (MoS_2_) as a thin layer and microparticles. Also, allergy tests evaluated on guinea pig skin to examine the allergic effect. The results showed lower toxicity of MoS_2_ nano-structures to the biological medium when the mass is less than 0.016 mg mL^-1^ [30].

This article details a straightforward, low-cost approach that employs an aqueous hydrothermal method for synthesizing two-dimensional molybdenum disulfide (MoS_2_) nanocrystals and their potential applications to explore cytotoxicity, bioimaging, and cellular uptake of A549 cancer cells. The high-resolution transmission electron microscopy (HRTEM) and atomic force microscopy (AFM) results revealed that the sizes of the as-grown polydisperse MoS_2_ nanocrystals range between 2 and 5 nm; their corresponding thicknesses were verified to lie between 1 and 2 nm, a shred of clear evidence that a few-layer of MoS_2_ nanocrystals have been synthesized. Photoluminescence (PL) and time-resolved PL spectra for the MoS_2_ nanocrystals exhibited a strong emission in the blue region with a further slow decay constant.

Hence, in this report, the human lung carcinoma epithelial cell line (A549) after 24 hours exposure to the MoS_2_ nanocrystals was estimated and interpreted by applying the methyl-thiazolyl diphenyl-tetrazolium bromide (MTT) and water-soluble trypan blue assays. A549 cell line was favourably preferred for this research because the lungs are expected to be the first place in which TMD occupies and communicates with the whole body when breathed into the respiratory tract. MTT and trypan blue assays are founded cell viability assays that act in the same way. The number of viable cells after treating with the MoS_2_ will be comparable to the formation product’s color intensity. By using both MTT and trypan blue assays in our research, we could be convinced that the cytotoxicity results are assured if the order collected from each assay were consistent and complemented each other. In this direction, we analyzed the sensitivity of A549 cells to the tested nanomaterials. Cell viability was monitored using blue trypan and MTT assays. Reactive oxygen species (ROS) formation produced by MoS_2_ nanocrystals was also studied. Our research is also based on morphologic studies with the use of microscopic study.

## Experimental details

### Materials and reagents

Sodium molybdate dihydrate (Na_2_MoO_4_.2H_2_O) and thioacetamide (CH_3_CSNH_2_) were purchased from Sigma Aldrich. A549 Cell lines (a human alveolar epithelial cell line) was procured from the American Type Cell Culture (ATCC).

### Synthesis of MoS_2_ nanocrystals

All the chemicals applied in the study were scientific-grade and used as such. The synthesis procedure details are as follows: 0.8 g (3.3 mMol) of sodium molybdate dihydrate (Na_2_MoO_4_.2H_2_O) was added into 50 ml of deionized water, and then 0.7 g (9.31 mMol) of thioacetamide (CH_3_CSNH_2_) was mixed into the aqueous solution while stirring at room temperature. The solution mixture was carried into a Teflon-lined stainless-steel autoclave loaded with the aqueous solution up to 60% of the full capacity, then sealed and kept at 200°C for 24 hours. The collected black precipitates were centrifuged, cleaned with distilled water and ethanol five times, and then dried inside a vacuum oven at 60°C for 12 hours.

### Characterization details

The UV-2401(Shimadzu Corporation) spectrophotometer was used to study the absorption spectra of as-synthesized nanocrystal and bulk powder. The crystal structure of the bulk powder and as-grown nanocrystal was examined through the Rigaku Miniflex diffractometer with typical X-ray tube (Cu Kα radiation, 40 KV, 30 mA) and Hypix-400 MF 2D hybrid pixel array detector (HPAD) and the corresponding structure obtained from the analysis by High-score plus software. The size distribution and morphology of MoS_2_ nanocrystals were checked in the non-contact mode by Park XE-70 atomic force microscope (AFM). Structural analysis was carried out using a transmission electron microscope (TEM) (Model JEOL JEM-2100F) performed at accelerating voltage 200 kV. TEM analysis was made by drop casting the diluted MoS_2_ dispersion over the carbon-coated copper grid, followed by proper drying. The Raman spectra of the MoS_2_ nanocrystal was taken with the Renishaw Raman microscopes help using 532 nm (0.3 mW) laser, 10-second scans acquired with the laser 20-x objective of an Olympus microscope. PL spectra were obtained with Fluromax 4C HORIBA Scientific Spectro-fluorometer upon excitation of a spectrum of wavelengths using 450 W Xe lamp. The lifetime analysis was carried using the same HORIBA equipment with a PPD detector and nano led-320 excitation source (peak wavelength: 321 nm, pulse duration < 1.0 ns), and the result was interpreted using Data Station software. PL decay profile was obtained by time-correlated single-photon counting (TCSPC) method to know the recombination mechanism of photo-excited charge carriers.

### Cell culture

A549 cells were cultured in a humidified incubator at 37°C and 5% CO_2_ and maintained in RPMI 1640 culture medium supplemented with 2 mM glutamine, 4.5g glucose per litre, 10 mM HEPES buffer pH 7.2, gentamycin (10 μg/ml), and fetal bovine serum (10% V/V).

### In vitro cell viability assay

A549 cells [0.3×10^6^/ml/well] were seeded in triplicate in 24 well culture plates, treated with 5, 10 and 20 μg/ml of MoS_2_ for 24 hours, cells were harvested by trypsinization, and recoveries of trypan blue excluding viable cells proportion in the cell population was analyzed by cell counting using a hemocytometer. For MTT assay, cells [1×10^4^/ml/well] were seeded in a 96 well plate in triplicate for 24 hours and grown to 70 to 80% confluence. The cells were then incubated with fresh media containing 5, 10, and 20 μg/ml of MoS_2_ for 24 hours. Cells with only RPMI media served as the negative control. Following treatment, the cells were incubated with MTT (20 *μ*L/well from 5 mg/mL stock) for 4 h. Mitochondrial dehydrogenases of viable cells reduce the yellowish water-soluble MTT to water-insoluble formazan crystals, which were solubilized with the addition of DMSO. The medium was then removed, and 150 *μ*L of DMSO was added into each well to dissolve formazan crystals. Cell viability was measured by MTT assay, and absorbance was recorded at 560 nm.

### Reactive oxygen species (ROS) measurement

To detect the production of ROS, A549 cells [0.5 ×10^6^/ml/well] were cultured in each well of a 6 well plate, treated with or without 10 and 20 μg/ml of MoS_2_ for 24 hours. Cells were washed with PBS twice and incubated with 3 μM of H2DCFDA dye in PBS for 30 minutes in the dark at 37 ^0^C. Production of ROS inside the A549 cell in response to MoS_2_ was analyzed by NIS-Nikon fluorescence microscopy at 10X magnification.

### Cellular uptake MoS_2_ by A549 cells

To study the uptake of MoS_2_, cells [0.3 ×10^6^/ml/well] were cultured in each well of 12 well plates; after 24 hours of incubation, the cells were treated with or without 5, 10, and 20 μg/ml of MoS_2_ for 24 hours respectively. Then cells were trypsinized, washed twice with PBS, and the uptake was evaluated on a flow cytometer, BD FACS Verse.

### Statistical analysis

All experiments were executed in triplicate, and results were expressed in Mean± SEM.

## Results and discussion

**Fig 1:**
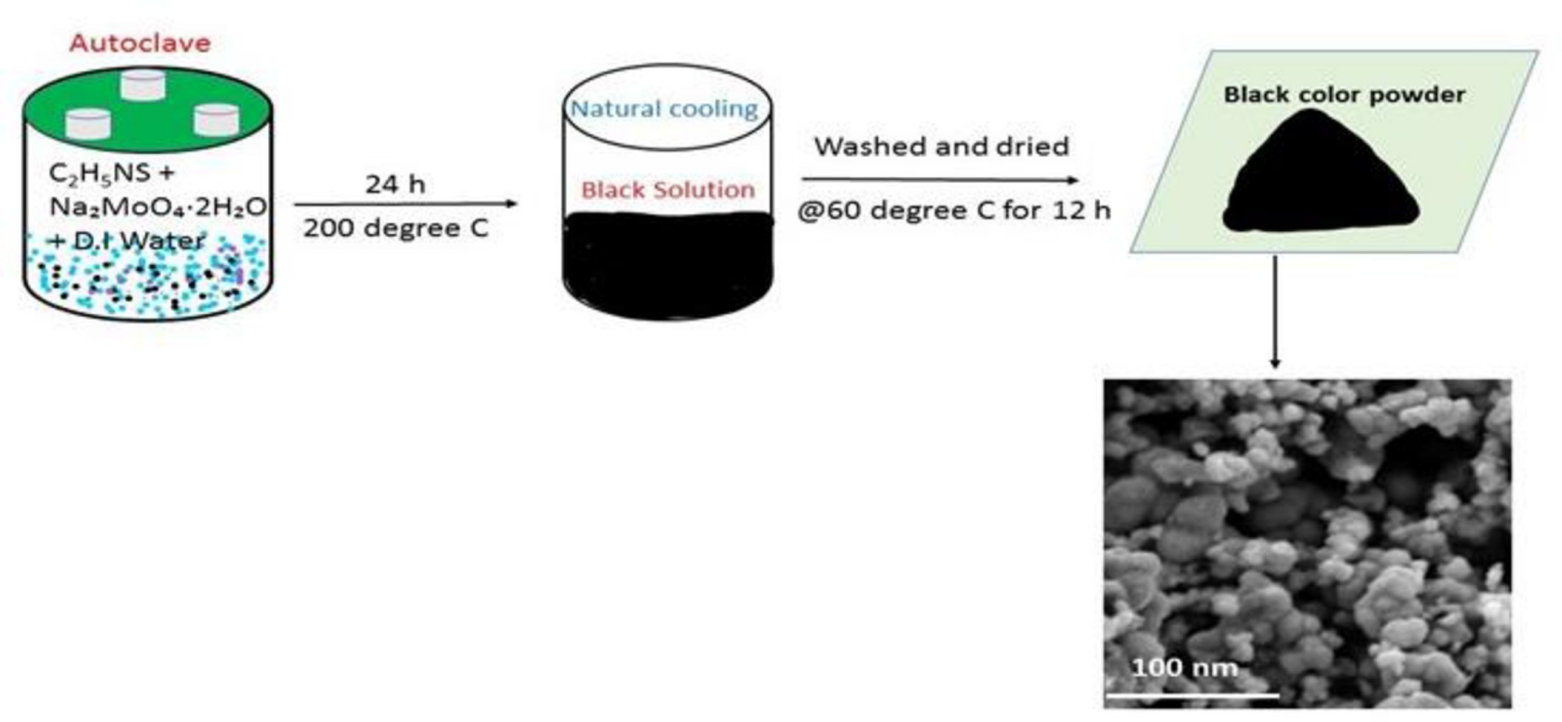
Schematic illustration of the steps for synthesis of spherical MoS_2_ nanocrystals.

**Fig 2:**
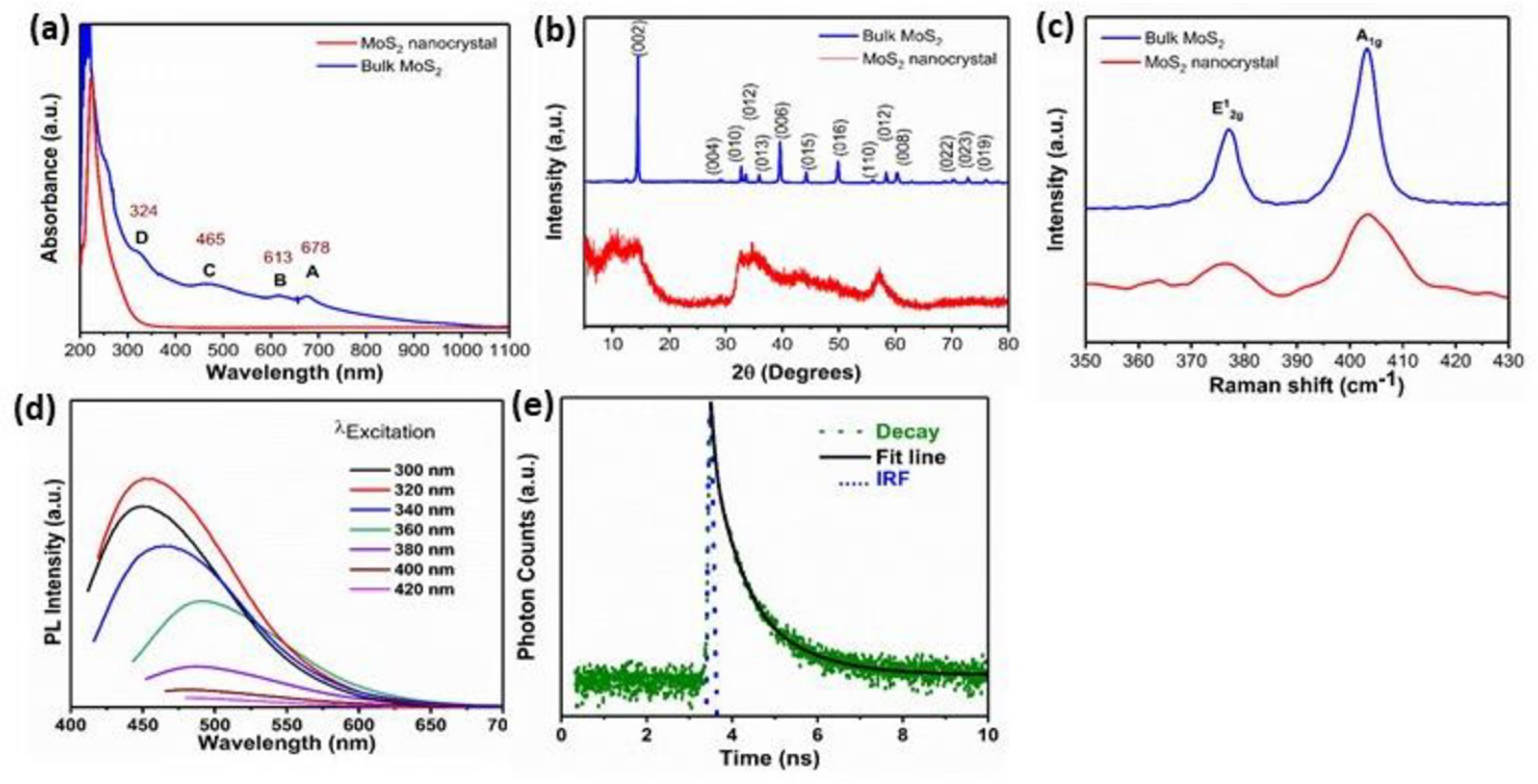
(a) UV–vis spectra of the as-prepared MoS_2_ nanocrystals and bulk MoS_2_. (b) XRD spectra (c) Raman spectra (d) Emission PL spectra of the as-prepared MoS_2_ nanocrystals under different excitation wavelengths (e) Fluorescence lifetime of MoS_2_ nanocrystals. The data are fitted using a tri-exponential decay model (black line) (f) Table shows PL lifetime (τ_1_, τ_2_ and τ_3_), and amplitude corresponding to different lifetime.

MoS_2_ nano-crystals sample was prepared using a one-step hydrothermal method wherein sodium molybdate and thioacetamide were used as sources of molybdate and Sulphur, respectively. The overall synthesis methodology is shown in Fig 1. The synthesis process is outlined in the experimental segment. To probe the optical properties, the UV absorption spectra of the nanocrystals and the bulk MoS_2_ were studied (Fig 2(A)). The excitonic peaks at positions A and B show the direct band-to-band transition at the K-point of the Brillouin zone. Furthermore, C and D peaks show the direct transition from the split valence band to the conduction band at the Brillouin zone’s M-point. The energy splitting within various absorbance peaks ("A & B" and "C & D") in the bulk MoS_2_ results from spin-orbit coupling and inter-player coupling. The energy splitting rises steadily with the reduction of layers number starting from the bulk sample. The absorption spectra of MoS_2_ nanocrystal with a strong excitonic peak near the ultraviolet regime at 224 nm confirm the synthesis of a few nanometer particles [31]. The crystal structure of bulk and nanocrystal MoS_2_ was studied using the X-ray diffraction (XRD) technique, as shown in Fig 2(B). The XRD spectra of bulk 2H-MoS_2_ show an intense peak at 2θ = 14.4°, which is assigned to (002) plane, along with other diffraction peaks, respectively (JCPDF-00-037-1492). Notably, the peak of the (002) position of MoS_2_ nanocrystals is becoming broad, symbolizing the lateral size reduction of the nanocrystals [32]. The reduction of intensity at (002) peak along the c-axis shows that the nanocrystals are few layers and too thin to be identified by XRD, which is exactly matching with the AFM and TEM results. Fig 2(C) reveals the characteristic Raman spectra of bulk powder and as-synthesized nanocrystals. The E_2g_^1^ mode appears from the in-plane vibrations of two S atoms with respect to the Mo atom, and A_1g_ mode results from out of plane vibration of S atoms only. The frequency difference (Δk) between the two Raman modes gives an idea about layer thickness. It has been seen that the Δk value for synthesized nanocrystals decreases to 24 cm^-1^ as compared to bulk MoS_2_ powder having a Δk value of 26 cm^-1^ [15,33]. However, the quantum size effect is accountable for tuning 2D TMDs nanocrystal’s optical properties. The PL emission spectra of as-synthesized MoS_2_ nanocrystals were studied under different excitation wavelengths ranging from 300 to 420 nm as shown in Fig 2(D). PL in MoS_2_ nanocrystals arises due to the excitation recombination at the electron or hole trap formed by uncompensated positive or negative charge at the dangling bond. An intense emission peak is observed at 460 nm under an excitation wavelength of 320 nm, while the intensity of emission spectra is continuously reduced and redshifted with a further increase of excitation wavelength. Here, the excitation-dependent PL measurements prove the poly-dispersive nature of MoS_2_ nanocrystals. The excitation-dependent spectra indicate polydispersity of the MoS_2_ nanocrystals distributions, which is vital of excitation recombination at the electron (hole) trap formed by the uncompensated positive (negative) charge at the dangling bond from as-grown MoS_2_ nanocrystals. This excitation-dependent PL response of fluorescent nanocrystals is useful for multicolor imaging purposes. As observed in earlier reports, the photoluminescence features of MoS_2_ nanocrystals is proportional to their particle dimension, which is related to the quantum size-effect of semiconductor for nanocrystals. The red shift of the emission spectra is also observed because of the size effect. MoS_2_ nanocrystal products have excellent dispersal, small size and PL properties in aqueous suspension and have encouraged biomedical applications [34–36]. The PL decay curve of MoS_2_ nanocrystals is exhibited in Fig 2(E). The photoluminescence decay of the as-grown sample is performed using a 321 nm laser LED excitation source. Instrumental response function (IRF) (shown by the green dotted line) was recorded using dilute Ludox colloid to maximize Rayleigh scattering and decrease the scattering effect from impurities, cuvette, and solution. The emission monochromator was fixed to the same wavelength as the excitation source (321 nm), and both polarizers were set to perpendicular to measure the IRF. It has been fitted with a third-order exponential equation I=I0+A1e−tτ1+A2e−tτ2+A3e−tτ3 with an average reduced weighted residual value of <1.2, and the fitted curve (solid red and pink line) convoluted with IRF is shown in Fig 2(E).

The curve is fitted with a third-order exponential function, appearing in three governing excitonic phenomena accompanied by nanosecond luminescence lifetime. The average PL lifetime of the as-prepared MoS_2_ nanocrystals was determined to be 5.615 ns. The increase in nanocrystals’ average PL lifetime is due to the defect state formation during synthesis [12,31].The collected lifetime (*τ*_1_, *τ*_2_ and *τ*_3_) and amplitude of i_th_ lifetime components (A_i_) are shown in Table 1.

**Table 1:** The collected lifetime (*τ*_1_, *τ*_2_ and *τ*_3_) and amplitude of ith lifetime components (A_i_).

*τ*_1_(ns)	A_1_	*τ*_2_(ns)	A_2_	*τ*_3_(ns)	A_3_	〈τ〉(ns)
3.612	47.32	0.582	15.45	10.253	37.22	5.615

**Fig 3:**
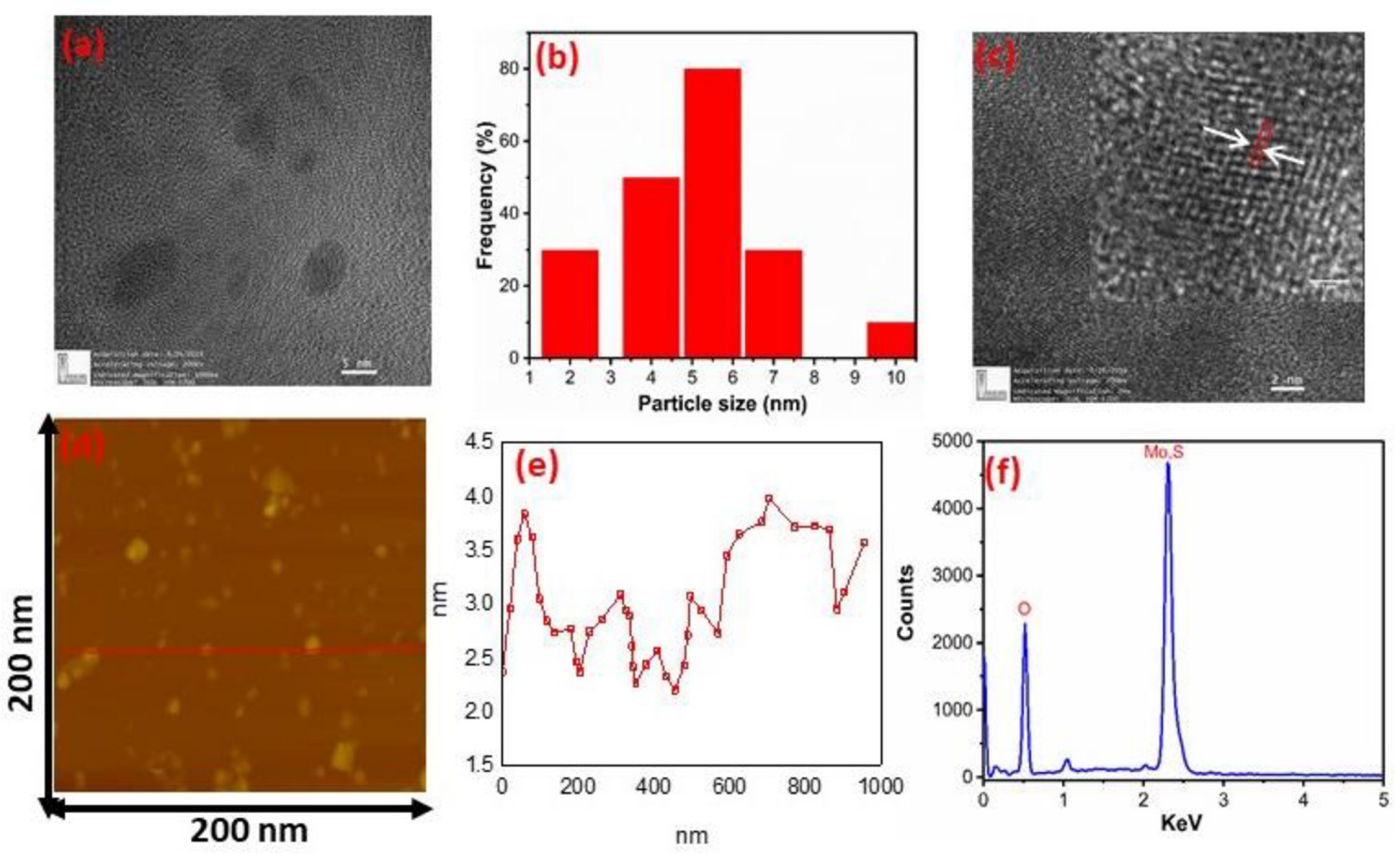
(a) HRTEM image of MoS2 nanocrystals (b) shows the size distribution (c) show the lattice fringes (d) Corresponding AFM image of nanocrystals (e) Height profile corresponding the line in d and (e) EDAX spectrum of MoS2 nanocrystals.

Fig 3(A) shows the HR-TEM image of MoS2 nanocrystals presenting the hexagonal lattice structure. The MoS2 nanocrystals with diameters of 2–10 nm are uniformly distributed; the size distribution of MoS2 nanocrystals is sketched and shown in Fig 3(B). The high-crystalline nature of the nanocrystals with lattice spacing of 0.2 nm matching to the very clear lattice fringes along with the (006) directions (shown in inset of Fig 3(C)), this is indicative of the high crystalline order of the nanocrystals. The atomic force microscopy (AFM) image was obtained to identify the morphology and the thickness distribution of the MoS2 nanocrystals, as shown in Fig 3(D) and 3(E) respectively. The nanocrystal thickness ranges from 1nm to 4 nm, indicating that the synthesized nanocrystals are of few-layer. The composition of as-prepared MoS2 samples was studied by Energy-dispersive X-ray spectroscopy (EDAX). Fig 3(F) exhibits the EDAX image of MoS2 nanocrystals. The EDAX study proved that the as-synthesized MoS2 nanostructure comprises Mo and S elements, including oxygen atoms. Further, no other elements were found, which verified the purity of as-prepared samples [1,12,32].

### Effect of MoS_2_ on cell viability

**Fig 4:**
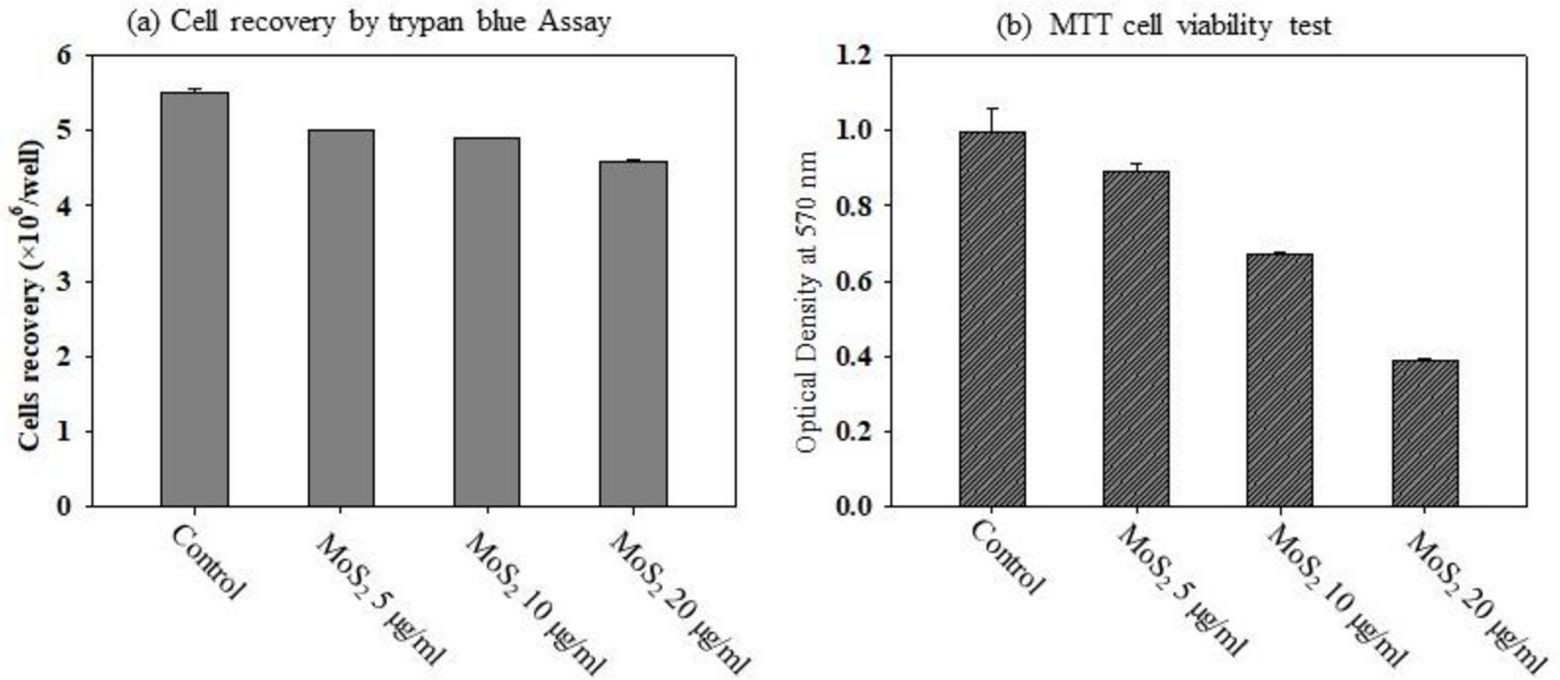
(A) Trypan blue exclusion and (B) MTT assay were used to assess the effects of MoS_2_ nanocrystals on A549 cells viability.

For the cytotoxic effect of MoS_2_, trypan blue cell exclusion dye and the MTT colorimetric test were used. After incubation of 5,10, 20 μg/ml of MoS_2_ for 24 hours, using the trypan blue method, it is observed that MoS_2_ did not produce any significant cytotoxic effect on the A549 cell viability up to 10 μg/ml of concentration. However, there was a significant decline in the number of viable cells (Fig 4(A)) in the highest concentration 20 μg/ml MoS_2_ used, compared to the control cells. For further confirmation, the effect of MoS_2_ on the cell viability of A549 cells by MTT assay was investigated. The result showed a concentration-dependent toxic profile with maximum toxicity observed at the highest concentration used, a 60% reduction in cell viability, which corresponds to the decrease in the absorbance measurement, as shown in Fig 4(B). Therefore, the two cytotoxic tests confirmed the toxic response of MoS_2_ to higher doses on the cell viability of A549 cells.

### Effect of MoS_2_ on the production of ROS in A549 cells

**Fig 5:**
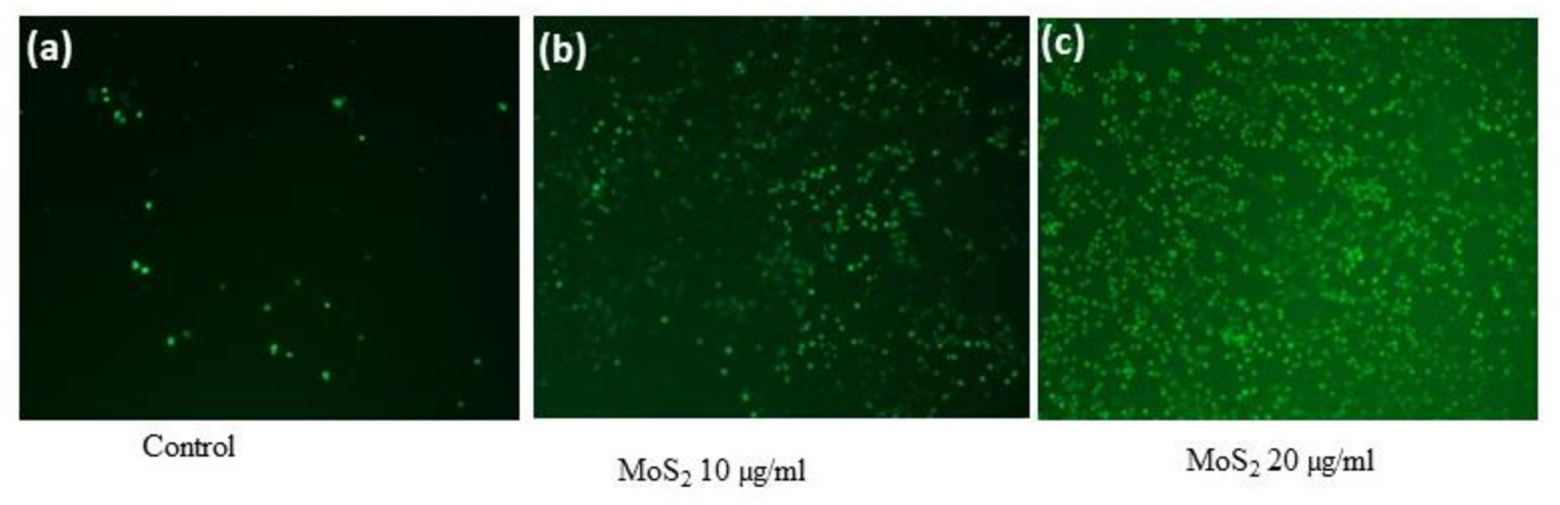
Effect of MoS_2_ nanocrystals on ROS production in A549 cells (a) Control (b) 10 μg/ml and (c) 20 μg/ml.

Several studies have suggested that ROS generation and oxidative stress production may be one of the underlying mechanisms, leading to nanoparticle-induced cytotoxicity in different cell types [37–40]. We analyzed the formation of reactive oxygen species in response to MoS_2_, and found a significant production of ROS in cells at higher doses of MoS_2_ (Fig 5(A)–5(C)). These results are also correlated with our result of the cytotoxic effect of particles and could be the cause of cell death seen at higher doses.

### Cellular uptake of MoS_2_ by A549 cells

**Fig 6:**
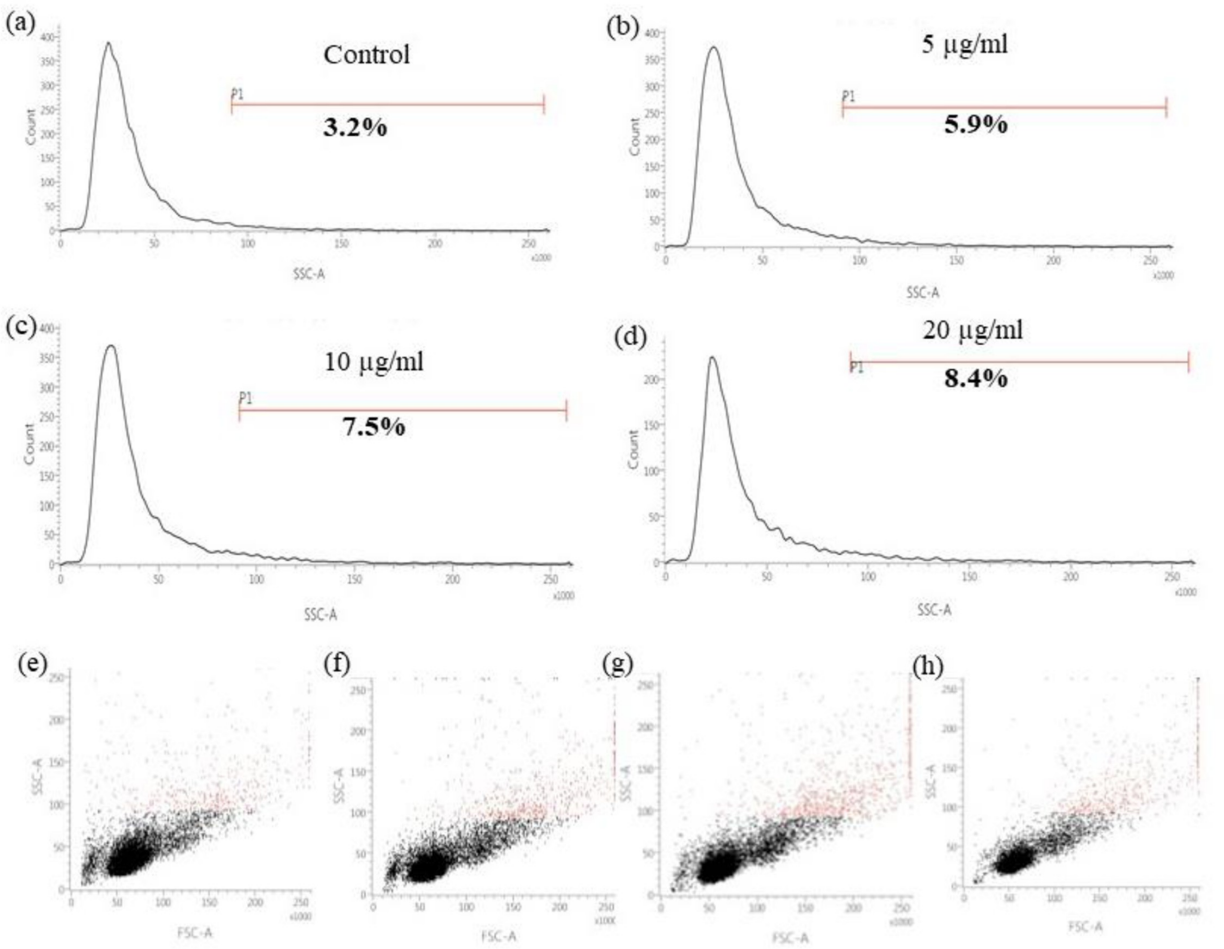
Uptake of MoS2 nanoparticles by A549 cells. Side scatter (SSC) measure of (a) control, (b) 5 μg/ml, (c) 10 μg/ml and (d) 20 μg/ml subjects under flow cytometry. Flow cytometry analysis of the dot plot of mean SSC value distribution of control and different concentration of MoS_2_ (e) control, (f) 5 μg/ml, (g) 10 μg/ml and (h) 20 μg/ml respectively.

To study MoS_2_ uptake by A549 cells, flow cytometry was used after cellular incubation with MoS_2_. The side scatters (SSC) value of flow cytometry reflects the evaluation at a 90° angle and correlates with the cell’s concentration. It has been studied that the value of SSC is correlated with complexity and internalized nanoparticles. Therefore, we compared the SSC value of both control and MoS_2_ treated cells and observed an increase in the SSC value of cells treated with different concentrations of MoS_2_ with respect to control cells (Fig 6(A)–6(D) and Table 2). A significant change in the SSC signal was recorded in the treated cells corresponding to the control (Fig 6(E)–6(H)). This increase in the SSC value is significantly higher (8.4% for 20 μg/ml concentration) than control (3.2%) at the highest doses used in the study. These findings indicate that the MoS_2_ nanoparticles could be taken up by the A549 cells and internalized inside the cells, causing the generation of ROS and affecting cell viability. However, further study is needed to confirm a detailed picture of the internalization and localization of MoS_2_ inside the cells and how exactly it is interfering and modulating the cell behaviour.

**Table 2:** SSC-A mean value of A549 cells after 24 h incubation with MoS_2_.

Dosimetry	Control	MoS_2_ 5 μg/ml	MoS_2_ 10 μg/ml	MoS_2_ 20 μg/ml
A549 cells	114,313	116,970	120,697	121,444

## Conclusion

This study used a one-step, bottom-up, hydrothermal route to synthesize blue luminescence MoS_2_ nanocrystals using sodium molybdate dihydrate (Na_2_MoO_4_.2H_2_O) and thioacetamide (CH_3_CSNH_2_) as precursors. The as-prepared MoS_2_ nanocrystals show a small lateral size distribution. Complete microscopic and spectroscopic techniques, including TEM, EDAX, AFM, XRD, UV-Vis, PL, TRPL, and Raman spectroscopy, were employed to confirm the morphology and composition of the MoS_2_ nanocrystals. The PL properties, linked with the adequate biocompatibility and physiological stability of MoS_2_ nanocrystals, directed to suitable bioimaging performance. Finally, cell viability measurements were performed with MTT and trypan blue assays after exposing human lung epithelial cell (A549) culture with different concentration of MoS_2_ nanocrystals for the duration of 24 h. Treatment of A549 cells with MoS_2_ nanocrystals caused a dose-dependent increase in ROS formation up to 20 μg/ml. Eventually, as the toxicity studies of MoS_2_ nanocrystals are still in its start, further study will be needed from the scientific societies to resolve their health impacts in the long period and assure that the potential hazards are estimated before incorporating the MoS_2_ nanocrystals into several biomedical application.
